# Microvascular endothelial function in Japanese early adolescents

**DOI:** 10.3164/jcbn.17-58

**Published:** 2017-10-19

**Authors:** Yutaka Odanaka, Kimitaka Takitani, Hiroshi Katayama, Hiroshi Fujiwara, Kanta Kishi, Noriyasu Ozaki, Atsuko Ashida, Ryuzo Takaya, Hiroshi Tamai

**Affiliations:** 1 Department of Pediatrics, Osaka Medical College, 2-7 Daigakumachi, Takatsuki, Osaka 569-8686, Japan; 2 Department of Pediatrics, Kyoto Prefectural University of Medicine, Kajii-cho, Kawaramachi-Hirokoji, Kamigyo-ku, Kyoto 602-8566, Japan; 3 Department of Pediatrics, Saiseikai Ibaraki Hospital, 2-1-45 Mitsukeyama Ibaraki, Osaka 567-0035, Japan

**Keywords:** endothelial function, peripheral arterial tonometry, early adolescents

## Abstract

Endothelial dysfunction is the early predictive factor for the development of atherosclerosis and future cardiovascular diseases in adulthood. The prevalence of endothelial dysfunction in children and early adolescents is increasing worldwide. Peripheral arterial tonometry is a noninvasive technique for assessing peripheral microvascular function and is used as a validated marker of endothelial function. We assessed anthropometric parameters, blood pressure, arterial stiffness, and peripheral endothelial function in 157 Japanese early adolescents (75 boys and 82 girls). We measured peripheral endothelial function by using peripheral arterial tonometry to determine the reactive hyperemia index, and assessed the association of reactive hyperemia index with parameters of anthropometry and arterial stiffness. The mean reactive hyperemia index of all subjects was 1.85 ± 0.6, and there was no difference of reactive hyperemia index according to sex. Reactive hyperemia index was significantly associated with systolic and diastolic blood pressures, and had no correlation with anthropometric parameters and arterial stiffness markers. The reactive hyperemia index values among Japanese early adolescents were similar to those reported in previous studies on children and early adolescents. This noninvasive technique may be useful for the assessment of microvascular endothelial function among children and early adolescents.

## Introduction

Endothelial cells lining the inner surface of vessels in a single layer constitute the endothelium and have a critical role in the homeostasis of the vascular system.^(1–3)^ The endothelium regulates vascular tone by generating and releasing a variety of vasodilating substances including nitric oxide, endothelin-derived hyperpolarizing factor, and prostacyclin, as well as vasoconstrictors such as endothelin and vasoconstrictor prostanoids.^(1–3)^ Moreover, the endothelium inhibits smooth muscle growth, controls leukocyte adhesion, regulates inflammation, and promotes antithrombotic activity.^(4)^ Endothelial dysfunction is characterized by impairment of the above-mentioned functions of the endothelium due to several risk factors including hypertension, dyslipidemia, diabetes, alcohol addiction, and smoking.^(2,5)^ Therefore, endothelial dysfunction is the early predictive factor for the development of atherosclerosis and future cardiovascular diseases in adults.^(2)^ Recently, the prevalence of endothelial dysfunction among children and early adolescents is increasing worldwide with the increased prevalence of risk factors for cardiovascular diseases, including obesity, hypertension, insulin resistance and dyslipidemia.^(6)^

For assessing peripheral endothelial function, various noninvasive techniques have been developed.^(7)^ Flow-mediated dilatation (FMD) of the arm arteries is the most universally applied noninvasive method to measure endothelial function, and evaluates the arterial ability in response to reactive hyperemia.^(8)^ Therefore, among children and young adolescents, the assessment of endothelial function with FMD is widely conducted to detect and monitor the progression of subclinical atherosclerosis. However, FMD has two major concerns: dependence on an ultrasound operator and need for considerable practice and standardization. Peripheral arterial tonometry (PAT) has been developed as an alternative technique to FMD in evaluating endothelial function, and is based on changes of digital pulse volumes during reactive hyperemia.^(9)^ PAT, which is a noninvasive method for assessing peripheral microvascular function, reflects coronary microvascular function in subjects with early-stage atherosclerosis and is a predictor of cardiovascular events.^(10)^ The reactive hyperemia index (RHI) is calculated by using a computerized algorithm, and is used as a validated marker of endothelial function.^(8,11)^ However, conflicting results exist with respect to the correlation between FMD and PAT. It is likely that these two methods evaluate different functions of the macrovasculature and microvasculature, respectively.^(9)^

The repeatability and reliability of PAT for evaluating endothelial function is proven among children and adolescents,^(12)^ and the number of clinical studies elucidating endothelial function by using PAT for children and adolescents are increasing yearly.^(13)^ Several disorders affect microvascular endothelial function from childhood to adolescence as well as in adulthood. The RHI of survivors with acute lymphoblastic leukemia was lowered compared with that of control subjects.^(14)^ There are conflicting results about the RHI of obese early adolescents.^(15–18)^ The RHI of early adolescents with obesity is either decreased or did not change compared with that of healthy subjects. However, the RHI of obese early adolescents was improved by diet and exercise.^(19)^ Adolescents with Kawasaki disease or Fontan circulation showed lower RHI than healthy controls.^(20,21)^ The microvascular endothelial function of adolescents with type 1 diabetes or Crohn’s disease was significantly decreased than that of healthy controls.^(22,23)^

There are a number of reports about the assessment of RHI in children and adolescents with various disorders, as mentioned above; however, to our knowledge, few studies have evaluated RHI in Asian children. The aim of this study was to evaluate the changes of RHI according to age and sex differences, and the association of RHI with parameters of anthropometry and arterial stiffness among early adolescents in Japan.

## Subjects and Methods

### Subjects

In the current study, we enrolled 157 healthy young adolescents (75 boys and 82 girls; age, 13.7 ± 0.9 years) from two junior high schools in Okayama, Japan, in October 2014 and 2015. The subjects had no diabetes, dyslipidemia, hypertension and renal diseases, and no history of vasculitis including Kawasaki disease, Raynaud’s disease, and juvenile idiopathic arthritis. None of the subjects smoked or had body mass index (BMI) >95th percentile. The study protocol was approved by the ethics committee of Osaka Medical College (code no. 1534), and informed consent for participation was obtained from both the subjects and their parents.

### Anthropometric measurements

With the subjects in light clothing, weight was measured to the nearest 0.1 kg on a digital weight scale and height was measured to the nearest 0.1 cm by using a standard stadiometer. Waist circumference was measured to the nearest 0.1 cm by using a plastic measuring tape at the level of the umbilicus while the subject was at minimal respiration. BMI was calculated as weight divided by height squared (kg/m^2^), and the BMI percentile was determined on the basis of population data from Japan.^(24)^ Blood pressure was measured by using an automatic sphygmomanometer. Body composition was evaluated by measuring the body fat percentage, which was calculated with a biochemical impedance analyzer, as previously described.^(25)^

### Arterial stiffness

Measurement of brachial-ankle pulse wave velocity (PWV), which is known as a noninvasive plethysmographic method for evaluating arterial stiffness, was conducted as previously described.^(26)^ The ankle-brachial index (ABI), which is the ratio of systolic blood pressure at the ankle to that in the upper arm, was evaluated concurrently.

### Endothelial function

To evaluate endothelial function, RHI was assessed by using the EndoPAT 2000 device (Itamar Medical, Caesarea, Israel) in accordance with the manufacturer’s instructions. Because measured values are affected by meals and caffeine intake, the enrolled subjects fasted overnight or had a light meal during the morning, and did not exercise intensively within 2 h before the measurement. All subjects were tested in the supine position while keeping quiet on a bed. Specialized pneumatic fingertip probes were placed on both fingers to measure pulse wave amplitudes throughout the protocol. After the measurement of pulse wave amplitudes at rest for 5 min, the arterial blood flow to one arm was occluded for 5 min by using a blood pressure cuff inflated to supra-systolic pressure (60 mmHg above systolic pressure or at least 200 mm Hg). After the 5-min occlusion, the cuff was immediately deflated, and finger arterial pulse wave amplitudes were measured for another 5 min. On the basis of the obtained data, a computerized algorithm of the software program automatically calculated the RHI.

### Statistical analyses

Statistical analyses were performed with JMP 11 (SAS Institute Inc., Cary, NC). Shapiro-Wilk tests were used to check data for normal distribution. Statistical comparisons between male and female subjects were analyzed by using a paired *t* test or the nonparametric Kruskal-Wallis signed-rank test, followed by the Mann-Whitney *U* test. The correlations with the RHI of each variable were evaluated with univariate and multiple analyses. All data are presented as mean ± SD, and *p*<0.05 was considered to indicate statistical significance.

## Results

### Subjects’ characteristics

The clinical characteristics of 157 healthy young adolescents are summarized in Table 1. No sex difference was observed in variables including age, BMI percentile, blood pressure, RHI, PWV and ABI. The height, weight and waist circumference of boys were significantly higher than those of girls, whereas the body fat percentage and heart rate of girls were higher than that of boys.

### Changes of RHI with age and sex

**Fig. 1:**
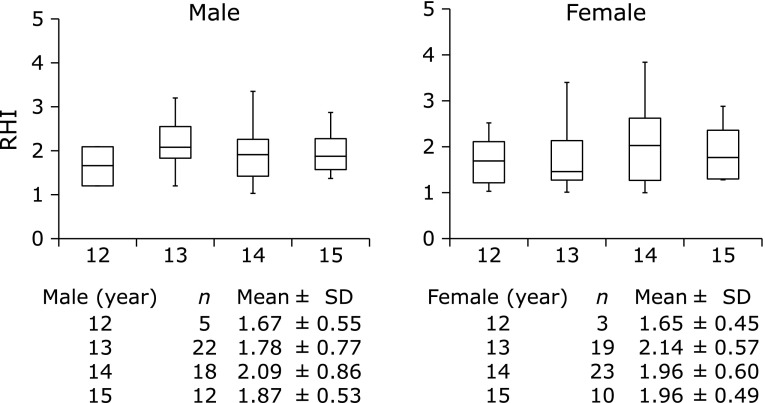
Change of reactive hyperemia index (RHI) with age among boys and girls. Number of subjects and mean ± SD by age are represented.

The median RHI values in all subjects, in boys and in girls were 1.85 ± 0.6, 1.82 ± 0.66 and 1.87 ± 0.54, respectively. There was no difference of RHI according to sex. The changes of RHI with age were divided by sex in Fig. 1. The mean RHI of boys at age 12, 13, 14 and 15 years was 1.67 ± 0.55, 1.78 ± 0.7, 2.09 ± 0.86 and 1.87 ± 0.53, respectively, and that of girls at age 12, 13, 14 and 15 years was 1.65 ± 0.45, 2.14 ± 0.57, 1.96 ± 0.6 and 1.96 ± 0.49, respectively. There was no difference of RHI between each age of boys and girls.

Relation between RHI and various parameters. Next, we analyzed the correlation of RHI with several parameters by using univariate analysis (Table 2). RHI was negatively correlated with systolic blood pressure (r = −2.48, *p* = 0.01) and diastolic blood pressure (r = −4.26, *p*<0.0001), and positively associated with left ABI (r = 2.12, *p* = 0.03). There was no significant difference between RHI and parameters including age, height, body weight, waist circumference, body fat percentage, BMI percentile and PWV. According to multiple correlation analysis, RHI had a markedly inverse correlation with systolic blood pressure (r = −2.13, *p* = 0.03) and diastolic blood pressure (r = −3.21, *p* = 0.001) (Table 3).

## Discussion

In the current study, we evaluated the RHI of Japanese early adolescents by using an Endo-PAT 2000 device. To our knowledge, this is the first report on the reference RHI in Asian early adolescents. The sample size of this study was relatively large compared with most of the prior studies, which had a sample size of less than approximately 50–100. Chen *et al.*^(27)^ reported that the RHI of Swedish healthy early adolescents (age, 14.5 ± 1.0 years, *n* = 250) was 1.7 (median, 1.4–2.0). Agarwal *et al.*^(17)^ demonstrated that the RHI was 1.98 ± 0.09 in 14 American control subjects (age, 14.93 ± 0.64 years), most of whom were Hispanic or African American. Moreover, the RHI of 44 Canadian boys (age, 14.2 ± 1.91 years) was 1.92 ± 1.23.^(28)^ Bonetti *et al.*^(29)^ proposed an RHI cutoff value of ≤1.35 among adults; however, there is no study with respect to the threshold of RHI among children and adolescents. In the present study, we revealed that the RHI of early adolescents was 1.85 ± 0.6, which corresponded closely to the data of the above-mentioned prior studies. The difference of RHI according to race among early adolescents is unlikely to be found.

Several studies demonstrated that RHI increases with age and pubertal development.^(30–33)^ In the current study, RHI was not associated with age in boys and girls; however, the RHI of both boys and girls at age 12 years was relatively lower than that of those aged 13 years or older. Moreover, we found no sex difference of RHI in Japanese early adolescents, which is in agreement with previous reports.^(18,32,34)^ The present study did not include children aged 12 years or younger; therefore, the association between RHI and age may be undetected. Kelly *et al.*^(30)^ speculated that the lower RHI of children than that of early adolescents is due to mechanical aspects related to the finger probes of the equipment. The probes are of only one size (designed for adults) and not sufficiently suitable for children. However, in the present study, the enrolled subjects were early adolescents; therefore, the finger probes of the equipment may be adequately appropriate for the sizes of their fingers. RHI is associated with the Tanner stage^(30,31)^ and significantly correlated with estrogen and dehydroepiandrosterone sulfate levels.^(33)^ These findings suggest that sexual hormones may play a critical role in endothelial function and the evolution of the vascular response.^(33)^ Pubertal development may affect RHI; therefore, the cutoff of RHI should be defined based on the degree of maturity rather than age specificity.^(13)^ However, we did not elucidate the correlation of RHI with Tanner stage and sexual hormone levels, which indicate sexual maturation or pubertal development. Further experiments on children and early adolescents with a broad age range or pubertal development will be required to clarify these issues.

Noninvasive modalities for the evaluation of endothelial function are useful for obese children and adolescents with subclinical atherosclerosis.^(6)^ The RHI of obese children and/or early adolescents is lower than that of healthy subjects.^(17,35,36)^ Bruyndonckx *et al.*^(19)^ reported that the RHI of obese adolescents improved with the therapeutic program composed of supervised diet and physical training. The association of RHI with BMI is inconsistent. RHI has been found to be positively associated with BMI in adolescents.^(17,31)^ In other studies, there was no correlation between RHI and BMI among obese and/or healthy adolescents.^(16,28,30)^ In the present study, the RHI of Japanese early adolescents was not correlated with the BMI percentile and body fat percentage. The body weight of most of the enrolled boys and girls was within the normal range. The reason for this finding is not clear; however, this relationship may vary according to body composition, age and race.

Hypertension is associated with endothelial dysfunction, which is promoted by decreased production of nitric oxide and enhanced oxidative stress.^(37)^ Hypertension enhances age-associated changes of vascular structure and endothelial function. Thus, blood pressure and endothelial function have a close relationship with respect to the maintenance of the vascular homeostasis. Concerning the association of RHI with blood pressure, there are paradoxical findings in studies on children and adolescents. RHI is positively associated with systolic blood pressure,^(28,30,31)^ whereas RHI has a negative association with systolic blood pressure^(16)^ or diastolic blood pressure.^(33,38)^ In a large-scale adult study in the United States, a lower prevalence of abnormal PAT ratio was associated with elevated systolic blood pressure.^(11)^ Moreover, in a Brazilian adult study, lower systolic blood pressure was associated with a lower PAT ratio.^(39)^ The current study revealed that RHI was inversely associated with both systolic and diastolic blood pressures. The reason for these conflicting findings is unclear; however, with respect to the pulse amplitude response to hyperemia, blood pressure may play a role as a confounding factor affecting digital microvascular compliance.^(11,28)^ Blood pressure during childhood varies across the ages; therefore, further longitudinal studies during this period will be required to clarify this issue.

Arterial stiffness is associated with existing cardiovascular risk factors and atherosclerotic diseases, and it is shown to be an independent and strong predictor of future cardiovascular events.^(40,41)^ PWV measurement, as a typical noninvasive method, is widely used to assess arterial stiffness in adults, and PWV becomes elevated with vascular dysfunction and arterial stiffness.^(6,42)^ Recently, PWV was evaluated among children with obesity, type 1 diabetes, primary snoring, Kawasaki disease, and coarctation of the aorta after operation for the assessment of arterial stiffness.^(6,43)^ Especially, obese children have enhanced arterial stiffening, which may lead to future cardiovascular disease risk.^(43)^ Niboshi *et al.*^(44)^ evaluated arterial stiffness including PWV and ABI among approximately 1,000 Japanese children and adolescents, and the values of PWV progressively increased with age in boys and girls, whereas the values of ABI were not altered. Moreover, they reported that the values of PWV among boys were higher than those in girls. The change of PWV and ABI with age in the current study was consistent with the findings of the above-mentioned study; however, the values of PWV in the present study had no sex difference. Although the reason for this conflicting finding is unclear, it may be due to the difference of sample size of the study.

Microvascular function has a close relation with arterial stiffness, and both of them are affected by several cardiovascular risk factors including obesity, hypertension, dyslipidemia, diabetes, and advancing age.^(45)^ In a study on obese and healthy children, Bruyndonckx *et al.*^(19)^ revealed that RHI was negatively associated with PWV, which is a finding similar to that of the above-mentioned adult study by Mitchell *et al.*^(45)^ In the current study, the RHI among Japanese healthy boys and girls had no relation with PWV. The cause of this discrepancy is yet to be determined; however, it may be due to aging and diseases (e.g., obesity). To resolve this issue, studies on children and adolescents with several diseases including obesity, dyslipidemia, or insulin resistance will be needed.

The current study should be viewed from the standpoint of several limitations. First, we did not evaluate blood biochemical markers of insulin resistance and dyslipidemia, which are closely related to endothelial function. However, the anthropometric parameters among most of the enrolled subjects were relatively within the reference range. Second, we did not assess puberty development, including Tanner stage classification and sexual hormones. During puberty, endothelial function is affected by sexual hormones, which may support the proposed mechanisms for a sex difference or an age-related change of the pulse amplitude response to hyperemia.^(33,46)^ Third, this current study is of a cross-sectional design, and thus, demonstrates the association but not the causal relationship.

In conclusion, we demonstrated that microvascular endothelial function measured with Endo-PAT in Japanese early adolescents had no sex difference and exhibited the negative association of RHI with blood pressure. These findings are consistent with prior studies for children and adolescents. We are convinced that this noninvasive technique may be useful for the assessment of microvascular endothelial function among children and adolescents with several diseases, including diabetes, congenital heart diseases, Kawasaki disease, obesity, hypertension, and metabolic syndrome, which are risk factors for the development of cardiovascular disease in adulthood. Further prospective and large-scale studies in Asian children with the above disorders will be required to clarify this issue.

## Figures and Tables

**Table 1:** The clinical characteristics for healthy adolescents

Mean ± SD, median (IQR)	Total (*n* = 157)	Male (*n* = 75)	Female (*n* = 82)	*p* value
Age	14 (1)	14 (2)	14 (1)	0.45
Height	160.0 ± 7.1	163.8 ± 6.7	156.4 ± 5.3	<0.0001
Weight	48.5 ± 6.3	50.5 ± 6.5	46.7 ± 5.5	0.0001
Waist circumference	66.0 ± 4.9	66.8 ± 4.7	65.2 ± 5.0	0.01
Body fat percentage	15.7 ± 4.8	12.3 ± 3.8	18.9 ± 3.0	<.0001
BMI percentage	38 ± 21	38 ± 22	38 ± 20	0.78
SBP	113 ± 13	114 ± 13	111 ± 12	0.12
DBP	64 ± 10	64 ± 10	64 ± 9	0.46
HR	70.5 ± 9.6	68.9 ± 8.5	72.0 ± 10.2	0.04
RHI	1.85 ± 0.60	1.82 ± 0.66	1.87 ± 0.54	0.27
R-PWV	909 ± 105	914 ± 119	904 ± 91	0.66
L-PWV	917 ± 108	919 ± 14	915 ± 93	0.94
R-ABI	1.02 ± 0.09	1.03 ± 0.09	1.02 ± 0.08	0.59
L-ABI	1.03 ± 0.10	1.03 ± 0.10	1.02 ± 0.10	0.38

PWV, brachial-ankle wave velocity; ABI, ankle brachial index; SBP, systolic BP; DBP, diastolic BP; HR, heart rate; IQR, interquartile range. Data were analyzed with ANOVA, the nonparametric Kruskal-Waillis signed-rank test, or the χ^2^ test.

**Table 2:** Significant determinants of RHI univariate correlation analysis

	r value	*p* value
Age	0.78	0.43
Height	–0.52	0.6
BW	0.12	0.9
Waist circumference	0.58	0.56
Body fat percentage	0.41	0.68
BMI percentile	0.29	0.76
SBP	–2.48	0.01
DBP	–4.26	<0.0001
HR	0.28	0.77
R-PWV	1.44	0.15
L-PWV	1.41	0.16
R-ABI	1.85	0.06
L-ABI	2.12	0.03

Abbreviations are listed in Table 1.

**Table 3:** Significant determinants of RHI multiple correlation analysis

	r value	*p* value
Age	0.75	0.45
Height	–0.09	0.92
Weight	0.29	0.77
Waist circumference	0.56	0.57
Body fat percentage	–0.36	0.71
BMI percentile	0.03	0.97
SBP	–2.13	0.03
DBP	–3.21	0.001
HR	1.42	0.16
R-PWV	–0.16	0.87
L-PWV	1.05	0.29
R-ABI	0.95	0.34
L-ABI	1.37	0.17

Abbreviations are listed in Table 1.

## Abbreviations

ABI: ankle-brachial index
BMI: body mass index
FMD: flow-mediated dilatation
PAT: peripheral arterial tonometry
PWV: brachial-ankle pulse wave velocity
RHI: reactive hyperemia index
